# Pharmacokinetic-Pharmacodynamic Analysis of Spiroindolone Analogs and KAE609 in a Murine Malaria Model

**DOI:** 10.1128/AAC.03274-14

**Published:** 2015-01-27

**Authors:** Suresh B. Lakshminarayana, Céline Freymond, Christoph Fischli, Jing Yu, Sebastian Weber, Anne Goh, Bryan K. S. Yeung, Paul C. Ho, Véronique Dartois, Thierry T. Diagana, Matthias Rottmann, Francesca Blasco

**Affiliations:** a Novartis Institute for Tropical Diseases, Singapore; b Swiss Tropical and Public Health Institute, Basel, Switzerland; c University of Basel, Basel, Switzerland; d Novartis Institutes for Biomedical Research, Cambridge, Massachusetts, USA; e Novartis Pharma, Basel, Switzerland; f Department of Pharmacy, National University of Singapore, Singapore

## Abstract

Limited information is available on the pharmacokinetic (PK) and pharmacodynamic (PD) parameters driving the efficacy of antimalarial drugs. Our objective in this study was to determine dose-response relationships of a panel of related spiroindolone analogs and identify the PK-PD index that correlates best with the efficacy of KAE609, a selected class representative. The dose-response efficacy studies were conducted in the Plasmodium berghei murine malaria model, and the relationship between dose and efficacy (i.e., reduction in parasitemia) was examined. All spiroindolone analogs studied displayed a maximum reduction in parasitemia, with 90% effective dose (ED_90_) values ranging between 6 and 38 mg/kg of body weight. Further, dose fractionation studies were conducted for KAE609, and the relationship between PK-PD indices and efficacy was analyzed. The PK-PD indices were calculated using the *in vitro* potency against P. berghei (2× the 99% inhibitory concentration [IC_99_]) as a threshold (TRE). The percentage of the time in which KAE609 plasma concentrations remained at >2× the IC_99_ within 48 h (%*T*_>TRE_) and the area under the concentration-time curve from 0 to 48 h (AUC_0–48_)/TRE ratio correlated well with parasite reduction (*R*^2^ = 0.97 and 0.95, respectively) but less so for the maximum concentration of drug in serum (*C*_max_)/TRE ratio (*R*^2^ = 0.88). The present results suggest that for KAE609 and, supposedly, for its analogs, the dosing regimens covering a *T*_>TRE_ of 100%, AUC_0–48_/TRE ratio of 587, and a *C*_max_/TRE ratio of 30 are likely to result in the maximum reduction in parasitemia in the P. berghei malaria mouse model. This information could be used to prioritize analogs within the same class of compounds and contribute to the design of efficacy studies, thereby facilitating early drug discovery and lead optimization programs.

## INTRODUCTION

Today, about 40% of the world's population lives in areas with a significant prevalence of malaria (1). The estimated worldwide annual death rate ranges from 660,000 (2) to 1.2 million (3). Widespread resistance against common antimalarials is responsible for the recent increase in malaria-related mortality (4). The fact that even artemisinin combination therapies exhibit delayed parasite clearance in patients emphasizes the need for new drugs with novel modes of action (5–8).

In modern antibiotic drug development, pharmacokinetic-pharmacodynamic (PK-PD) principles are applied to select doses and dosing regimens. The pharmacokinetic (PK) profiles obtained in animal models and human volunteers are combined with values of drug potency *in vitro* and efficacy in animal models to calculate PK-PD indices that inform rational trial design (9–11). Based on these PK-PD approaches, antibiotics have been classified as exerting concentration-dependent or time-dependent killing. The question of whether time or concentration drives the efficacy of a given drug has been used to enable the selection of dosing regimens that optimize clinical efficacy while suppressing the emergence of resistant organisms, as well as to determine clinically relevant susceptibility breakpoints (12). In contrast, many antimalarials in use today were developed and clinically tested before the modern era of rational dose selection based on PK-PD properties. The doses and dose regimens in initial clinical trials, including those for the newer and most effective artemisinin derivatives (13), were derived empirically before detailed studies of pharmacokinetics had been conducted and PK-PD relationships were established (14). Less-than-adequate doses, especially in children or pregnant women (15, 16), are thought to have contributed to the emergence of resistance to many clinically used antimalarial drugs (17).

In recent years, numerous clinical pharmacokinetic and therapeutic drug monitoring programs have been launched to assess the adequacy of drug doses and to model dose response in uncomplicated and severe malaria (18–20). Quantitative pharmacodynamic readouts, such as parasitemia, parasite clearance rates, and parasite clearance time, are used to assess the parasitological response to treatment in relation to clinical outcome (21, 22). Based on these advances and a better definition of clinical and parasitological responses (23), new drug combinations have been proposed (24, 25), and doses have been revised when required (14). Although a standardized relationship between parasitological responses *in vitro* and clinical outcome remains to be fully established (26), progress has been made in understanding the PK-PD relationship for standard antimalarial drugs (27). The field is clearly moving toward a rational selection of dose, dosing frequency, and duration of antimalarial treatment (28). In this context, we have undertaken the effort to evaluate the PK-PD relationship for a class of spiroindolones and, in particular, one selected analog currently in clinical development for the treatment of malaria.

Plasmodium falciparum and Plasmodium vivax are the parasites that cause blood- and liver-stage malaria infection in humans, respectively. Preclinical *in vivo* testing of antimalarial drug candidates often uses rodent-specific parasites, of which Plasmodium berghei provides a robust and reproducible model of parasitemia in mice (29). It has been used extensively to support drug discovery programs, following validation with a wide range of drugs that have proven to have clinical efficacy against P. falciparum malaria (30, 31). Upon inoculation of P. berghei parasites, the infection of untreated mice with P. berghei (ANKA strain) invariably takes a lethal course within 6 to 7 days (32). Hence, both survival and a reduction in parasitemia can be monitored to evaluate (i) the ability of a drug candidate to clear all viable parasites, as measured by the absence of recrudescence up to 30 days postinfection (i.e., “cure”), and (ii) drug-mediated reduction in parasitemia at the end of treatment.

During a whole-cell screening campaign for P. falciparum proliferation inhibitors, a series of spiroindolones was identified. Lead optimization efforts were further undertaken to improve their pharmacokinetic properties and *in vitro* potencies against P. falciparum (33). Spiroindolones act through a novel mechanism that disrupts intracellular Na^+^ homeostasis through the inhibition of the parasite non-sarco(endo)plasmic reticulum Ca^2+^ (non-SERCA) P-type ATPase PfATP4 (32, 34). This same parasiticidal mechanism across the series is further supported by the conserved enantiomer specificity (33). KAE609 is a spiroindolone product of the lead optimization efforts and is currently in clinical development for the treatment of P. falciparum and P. vivax malaria. We previously showed that the compound exhibits pharmacokinetic properties in rodents compatible with once-daily oral dosing in humans (32). In the murine P. berghei malaria model, a single oral dose of 100 mg/kg of body weight of KAE609 was sufficient to cure 100% of the animals. Thrice-daily oral doses of 50 mg/kg also afforded a complete cure, while a 50% cure rate was achieved with a single dose of 30 mg/kg. In light of these results, it was speculated that a sustained reduction in parasitemia could be achieved at low doses in humans (32).

Dose-response experiments were performed to investigate the relationship between dose and efficacy (i.e., reduction in parasitemia) across the spiroindolone series. In order to further characterize KAE609, the most promising of this new class of antimalarials, a classical dose fractionation approach was adopted to identify the PK-PD index that correlates best with a reduction in parasitemia in the P. berghei malaria murine model. Once such a PK-PD relationship is established, we propose that it be used to prioritize analogs within the same class of compounds and to contribute to the design of efficacy studies, thereby facilitating early drug discovery and lead optimization programs.

## MATERIALS AND METHODS

### Chemicals.

All spiroindolone analogs studied (see Fig. S1 in the supplemental material) were synthesized at the Novartis Institute for Tropical Diseases, as described elsewhere (33). Organic solvents (acetonitrile and methanol) were purchased from Merck, Darmstadt, Germany; ethanol and hydrochloric acid (HCl) were obtained from Fisher Scientific, Leicestershire, United Kingdom; polyethylene glycol 400 (PEG 400) was purchased from Acros Organics, NJ, USA; d-α-tocopheryl polyethylene glycol 1000 succinate (vitamin E TPGS) NF grade was obtained from Eastman, Anglesey, United Kingdom; Solutol HS15 was obtained from BASF, Ludwigshafen, Germany; and acetic acid, warfarin, and citrate acid buffer (prepared from anhydrous citric acid) were obtained from Sigma-Aldrich, St. Louis, MO, USA.

### *In vitro* antimalarial activities of spiroindolone analogs.

Isolates of P. falciparum were maintained using standard methods (35) in an atmosphere of 93% N_2_, 4% CO_2_, and 3% O_2_ at 37°C in complete medium (CM) (10.44 g/liter RPMI 1640, 5.94 g/liter HEPES, 5 g/liter AlbuMAX II, 50 mg/liter hypoxanthine, 2.1 g/liter sodium bicarbonate, and 100 mg/liter neomycin). Human erythrocytes served as the host cells. *In vitro* antimalarial activity was measured using the [^3^H]hypoxanthine incorporation assay (36) with strain NF54 of P. falciparum (obtained from Hoffmann-LaRoche Ltd.). The compounds were dissolved in dimethyl sulfoxide (DMSO) at a concentration of 10 mM, diluted in hypoxanthine-free culture medium, and titrated in duplicate over a 64-fold range in 96-well plates. Infected erythrocytes (0.3% final parasitemia and 1.25% final hematocrit) were added to the wells. After 16 h of incubation, 0.25 μCi of [^3^H]hypoxanthine was added per well, and the plates were incubated for an additional 8 h (in contrast to the conventional 48-h plus 24-h assay) to enable a direct comparison with the *in vitro* (*ex vivo*) P. berghei assay (see below). The parasites were harvested onto glass fiber filters, and radioactivity was measured using a BetaPlate liquid scintillation counter (Wallac, Zurich, Switzerland). All assays were repeated at least three times. The results were recorded and expressed as a percentage of the untreated controls. The 50% inhibitory concentrations (IC_50_s) were estimated by linear interpolation. In addition, for the PK-PD analysis of KAE609, an IC_99_ value was determined by transforming the data from a scintillation counter (Anscombe transformation) to account for Poissonian measurement error. The inhibitory concentrations conferring 50% (IC_50_), 90% (IC_90_), and 99% (IC_99_) growth reduction were inferred from fitting a four-parametric logistic function to the transformed data independent of the log-transformed concentrations. Each of the three replicates was performed on a distinct 96-well plate. This has been accounted for in the fitting procedure by means of a Bayesian hierarchical model. The software used for parameter inference was Stan (37).

For the *in vitro* (*ex vivo*) P. berghei maturation assays (38), heparinized blood from infected mice (P. berghei GFP ANKA malaria strain PbGFP_CON_, a donation from A. P. Waters and C. J. Janse, Leiden University, Leiden, the Netherlands) (39) was washed with 9 ml of hypoxanthine-free culture medium and diluted with hypoxanthine-free culture medium and red blood cells (RBCs) from uninfected mice to a hematocrit of 5% and a parasitemia of 0.3%. Serial compound dilutions were prepared in DMSO and distributed as described above. The infected erythrocytes (0.3% final parasitemia and 2.5% final hematocrit) were added into the wells. After the plates were incubated for 16 h, 0.25 μCi of [^3^H]hypoxanthine was added per well, and the plates were incubated for an additional 8 h. The parasites were harvested, and the *in vitro* (*ex vivo*) P. berghei IC_50_, IC_90_, and IC_99_ values were determined as described above.

### *In vivo* pharmacokinetic (PK) studies for spiroindolone analogs in CD-1 mice.

The Institutional Animal Care and Use Committee (IACUC) of the Novartis Institute for Tropical Diseases (NITD), registered with the Agri-Food and Veterinary authority (AVA), Government of Singapore, reviewed and approved all animal experimental protocols. For the *in vivo* PK studies, female CD-1 mice (25 to 30 g) were obtained from the Biological Resource Center, Biopolis, Singapore, and were randomly assigned to cages. The mice were allowed to acclimate before the initiation of the experiments. Feed and water were given *ad libitum*. The compounds were formulated at a concentration of 2.5 mg/ml for a dose of 25 mg/kg administered orally (p.o.) and at a concentration of 1 mg/ml for a dose of 5 mg/kg given intravenously (i.v.). The solution formulation for p.o. and i.v. dosing contained 10% ethanol, 30% PEG 400, and 60% 10% vitamin E TPGS. The blood samples from the mice were collected at 0.02 (for i.v. only), 0.08, 0.25, 0.5, 1, 2, 4, 8, 16, and 24 h postdosing. The dose proportionality studies were conducted with KAE609. Formulations containing 0.1, 0.5, and 10 mg/ml were prepared to support oral dosages of 1, 5, and 100 mg/kg, respectively. Additional samples were collected at 32 h and 48 h postdose for the 1- and 5-mg/kg dose groups and up to 72 h postdose for the 100-mg/kg dose group. Groups of three mice were used for each time point. Blood was centrifuged at 13,000 rpm for 7 min at 4°C, and plasma was harvested and stored at −20°C until analysis.

### Extraction and quantitation of spiroindolone analogs in plasma.

Plasma samples were processed by protein precipitation using acetonitrile-methanol-acetic acid (90:9.8:0.2) to recover both analytes and internal standard (warfarin) using an 8-to-1 extractant-to-plasma ratio for all the analogs studied except (+)-1 (6-to-1 ratio) and (+)-6 (3.6-to-1 ratio) (33). After vortexing and centrifuging the mixture, the supernatant was removed and 5 μl of sample analyzed. Analyte quantitation was performed by high-performance liquid chromatography coupled with tandem mass spectrometry (LC-MS/MS). Liquid chromatography was performed using an Agilent 1100 high-performance liquid chromatography (HPLC) system (Santa Clara, CA), with the Agilent Zorbax XDB-Phenyl (3.5 μm, 4.6 by 75 mm) column at an oven temperature of 35°C for all the analogs studied [except (+)-5, (+)-6, and (−)-6 at 45°C], coupled with a API3200 triple quadrupole mass spectrometer (Sciex Applied Biosystems, Foster City, CA) for all the analogs studied except (+)-1, (+)-5, (+)-6, and (−)-6 that were analyzed using a QTRAP 4000 triple quadrupole mass spectrometer (Applied Biosystems, Foster City, CA). Instrument control and data acquisition were performed using the Applied Biosystems software Analyst 1.4.2. The mobile phases used were A, water-acetic acid (99.8:0.2 [vol/vol]), and B, either acetonitrile-acetic acid (99.8:0.2 [vol/vol]) for (+)-1, (+)-5, and (−)-6 or methanol-acetic acid (99.8:0.2 [vol/vol]) for all the other analogs studied, using a gradient of 0 to 0.2 min (10% B), 0.2 to 1.8 min (10 to 80% B), 1.8 to 2.5 min (80% B), 2.5 to 2.51 min (80 to 10% B), and 2.51 to 6 min (10% B), with flow rate of 1.0 ml/min and a run time of 6 min. Under these conditions, the retention times of various compounds ranged from 3.2 to 4.1 min. Multiple-reaction monitoring (MRM) was combined with optimized MS parameters to maximize detection specificity and sensitivity. The most intense MRM transitions [338.3/295.1 for (+)-1, 370.1/327.0 for (+)-2, 370.0/326.9 for (+)-3, 354.1/310.8 for (+)-4, 354.1/311.2 for (+)-5, 372.1/329.1 for (+)-6, 388.0/331.8 for (+)-7, 372.1/316.1 for (−)-6, and 388/332 for (−)-7] were used for quantitation. Most compounds were analyzed using electrospray ionization in the negative mode, except (+)-1, which was analyzed using positive mode. The recovery of the compounds from plasma was good and consistent across the concentration range studied. The lower limits of quantification for different compounds ranged from 1.3 to 70 ng/ml in plasma. A calibration curve was freshly prepared and analyzed with every set of study samples. Intraday variability was established with triplicate quality control samples at three concentration levels. The results were accepted if relative standard deviation was <15%.

### Pharmacokinetic analysis.

The mean values of the compound concentrations in plasma were obtained from three animals at each time point and plotted against time to generate concentration-time profiles. The pharmacokinetic parameters were determined using WinNonlin Professional, version 5.0.1 (Pharsight, CA, USA), and by noncompartmental modeling using software model 200 for oral dosing and model 201 for intravenous dosing. The oral bioavailability (*F*) was calculated as the ratio between the area under the concentration-time curve from 0 to infinity (AUC_0–∞_) following oral administration and the AUC_0–∞_ following intravenous administration corrected for dose (*F* = AUC_p.o._ × dose_i.v._/AUC_i.v._ × dose_p.o_.).

### *In vivo* antimalarial efficacies of spiroindolone analogs in NMRI mice.

All *in vivo* studies conducted at the Swiss Tropical and Public Health Institute (TPH) adhered to local and national regulations of laboratory animal welfare in Switzerland (permission no. 1731). For *in vivo* efficacy, female (specific-pathogen-free) National Medical Research Institute (NMRI) mice were obtained from Janvier, Le Genest-Saint-Isle, France. Only mice without visible signs of disease were used for the study. Standard laboratory conditions were adopted for husbandry. *In vivo* antimalarial activity was assessed using groups of five female NMRI mice (20 to 22 g) intravenously infected on day zero with 2 × 10^7^ erythrocytes parasitized with P. berghei GFP ANKA malaria strain PbGFP_CON_ (a donation from A. P. Waters and C. J. Janse, Leiden University) (39). Untreated control mice typically died between day six and day seven postinfection. Experimental compounds were formulated in 10% ethanol, 30% PEG 400, and 60% of 10% vitamin E TPGS and were administered orally in a volume of 10 ml/kg. Dose-response efficacy studies were conducted for each spiroindolone analog. Doses of 2.5, 5, 10, 30, and 100 mg/kg were administered to groups of 5 mice each as a single dose at 24 h postinfection. Parasitemia, expressed as parasitized red blood cells (pRBCs) of >100 RBCs, was determined 72 h postinfection using standard flow cytometry techniques (39).

### Dose-response relationship analysis for spiroindolone analogs.

Data analysis was done using nonlinear mixed-effect modeling (NONMEM version VI 2.0), which analyzed the PD data from multiple dose groups obtained simultaneously from different experiments. Within NONMEM, first-order conditional estimation (FOCE) was the method used (40). The log-transformed dose and level of parasitemia were used for analysis. For each compound, a dose-response model was built, and the ED_90_ (effective dose lowering 90% of parasitemia) values were determined using equation 1 (41), where *P* is the parasitemia measured at any given dose, *P_max_* is the maximum level of parasitemia, *P_min_* is the minimum level of parasitemia, EZ_50_ is the dose required to produce 50% of the maximal effect, *s* is the Hill slope describing the steepness of the curve, and *x* is the dose (in mg/kg).

1 $$P=P_{\min\;\;}+\left\{[P_{\text{max}}-P_{\text{min}}]/\left[1+10^{(\text{log}{\text{EZ}}_{50}-\text{log}\;x)\times s}\right]\right\}$$

### *In vivo* PK and dose fractionation studies for KAE609 in NMRI mice.

For the *in vivo* PK studies, female NMRI mice (specific pathogen free) were obtained from Janvier, Le Genest-Saint-Isle, France. To investigate the influence of the disease status on the pharmacokinetics, a single oral dose (5.3 mg/kg) of KAE609 was given to both the infected and uninfected NMRI mice, and the PK parameters were determined. The infected mice were dosed at 24 h postinfection. The formulation, sampling times (0.25, 0.5, 1, 2, 4, 8, 24, and 48 h postdose), extraction, and analysis were similar to those in the method described above.

The dose fractionation studies were conducted with daily oral doses of 0.5, 1, 2, 4, and 8 mg/kg. The total daily doses were either administered as a single dose (24 h postinfection) or divided into two oral doses (BID) or three oral doses (TID) per day (Table 1). Four mice were used for each regimen, with the control mice receiving vehicle only. Parasitemia, expressed as parasitized red blood cells (pRBCs) of >100 RBCs, was determined at 72 h postinfection (48 h after the initiation of treatment with KAE609) using standard flow cytometry techniques (39).

**TABLE 1:** Dose fractionation and corresponding PK-PD indices and level of parasitemia for KAE609

Total dose^*a*^ (mg/kg)	Individual dose (mg/kg)	No. of doses	PK-PD indices^*b*^			Parasitemia (mean ± SD) (%)^*c*^
			*C*_max_/TRE	AUC_0–48_/TRE	%*T*_>TRE_	
8	8	1	29.95	513.68	99.9	0.15 ± 0.03
	4	2	19.4	414.93	99.8	0.18 ± 0.04
	2.67	3	16.44	383.23	99.8	0.16 ± 0.02
4	4	1	14.56	148.45	54.5	0.63 ± 0.57
	2	2	7.92	106.02	56.1	0.43 ± 0.38
	1.33	3	6.00	92.12	57.3	0.55 ± 0.28
2	2	1	6.93	45.07	23.0	5.64 ± 1.69
	1	2	3.37	30.66	22.7	6.48 ± 3.94
	0.67	3	2.24	25.18	22.3	10.83 ± 4.67
1	1	1	3.21	14.57	10.7	17.82 ± 3.05
	0.5	2	1.49	10.16	8.8	18.19 ± 6.81
	0.33	3	0.96	8.55	0	20.31 ± 5.71
0.5	0.5	1	1.45	4.98	4.3	28.82 ± 4.49
	0.25	2	0.67	3.75	0	38.28 ± 2.95
	0.17	3	0.43	3.34	0	35.37 ± 5.32

a The total daily dose was given as one, two (once every 12 h), or three (once every 8 h) equally divided doses over 24 h.

b *C*_max_/TRE, ratio of peak plasma concentration (*C*_max_) to the threshold (TRE = 2× the IC_99_); AUC_0–48_/TRE, ratio of the area under the concentration-time curve from 0 to 48 h (AUC_0–48_) to the threshold; %*T*_>TRE_, percentage of the 48-h period during which the total compound concentration exceeded the threshold.

c Parasitemia expressed as parasitized red blood cells (pRBCs) of >100 RBCs (determined 48 h posttreatment). The mean ± SD level of parasitemia for vehicle was 35.51% ± 3.86%.

### Pharmacokinetic modeling and simulation for KAE609.

Population PK modeling of the concentration-time data of KAE609 in mice was performed using naive pooling without intersubject variability using NONMEM version VI 2.0. A two-compartment pharmacokinetic model with first-order absorption and nonlinear elimination was built to fit the concentration-time data of KAE609 generated in (infected and healthy) NMRI mice at 5.3 mg/kg. In the model, clearance (CL) was described by equation 2, where *C*_p_ is compound plasma concentration, the maximum metabolic rate (*V*_max_)/*K_m_* ratio is the maximal clearance achievable when *C*_p_ is ≪*K_m_*, and *K_m_* corresponds to the compound concentration at which the clearance reached 50% of the *V*_max_/*K_m_*. The primary model parameters derived were plasma clearance (CL) (in liters/h/kg), intercompartmental clearance (*Q*) (in liters/h/kg), central and peripheral volume of distribution (*V*_c_ and *V*_p_, respectively) (in liters/kg), and the absorption rate constant (*K*_a_) (in h^−1^). The diagnostic plots were analyzed for closeness to and randomness along the line of identity on the observed versus predicted concentration plot, as well as randomness along the weighted residual zero line on the predicted concentration or time. The estimates were accepted when the plots showed no systematic pattern (42). Based on the estimated primary model parameters (*V*_max_, *K_m_*, *V*_c_, *V*_p_, Q, and *K*_a_), the plasma concentration-time profiles and secondary pharmacokinetic parameters, such as *C*_max_, AUC at different doses, and dosing regimens, were simulated using the Berkeley Madonna software (version 8.0.1) (Berkeley Madonna, University of California, Berkeley, CA.

2 $$\text{CL}=V_{\text{max}}/\left(K_{\text{m}}+C_{\text{p}}\right)$$

### PK-PD relationship analysis of KAE609 (dose fractionation study).

The concentration of compounds that inhibited 99% of P. berghei growth (IC_99_) was used to calculate the threshold (TRE = 2 × IC_99_) and the PK-PD indices. The *C*_max_/TRE was defined as the ratio of peak plasma concentration (*C*_max_) to the threshold (2 × IC_99_), the AUC_0–48_/TRE was defined as the ratio of the area under the concentration-time curve from 0 to 48 h (AUC_0–48_) to the threshold (ratio without dimensions), and the percent time over the threshold (%*T*_>TRE_) was defined as the percentage of the 48-h period during which the compound concentration exceeded the threshold (43). The *C*_max_/TRE, AUC_0–48_/TRE, and %*T*_>TRE_ at different doses and dosing regimens were calculated using the Berkeley Madonna Software (version 8.0.1) (Berkeley Madonna, University of California, Berkeley, Berkeley, CA).

The relationship between PK-PD indices (log-transformed *C*_max_/TRE, log-transformed AUC_0–48_/TRE, and %*T*_>TRE_) and log-transformed parasitemia was analyzed by nonlinear regression analysis. An exploratory analysis of the PK-PD data was done using various models, such as maximum effect (*E*_max_), sigmoid *E*_max_, and with and without slope. A sigmoidal dose-response (variable-slope) model without constants was fitted to the data. GraphPad Prism version 5.02 for Windows (GraphPad Software, San Diego, CA, USA) was the software used for this analysis. The model choice was guided initially by a visual inspection of the *y*-by-*x* plots and then by correlation analysis and by evaluating the standard error of the estimate. The parasitemia (*P*) measures at any value of each different PK-PD index are expressed in equation 1, where EZ_50_ (otherwise referred to as EC_50_) is the value of the specific PK-PD index (*C*_max_/TRE, AUC_0–48_/TRE, and %*T*_>TRE_, respectively) required to produce 50% of the maximal effect, *x* is any of the PK-PD indices, and *P*_max_, *P*_min_, and *s* are as described previously. The values of *x* corresponding to the maximum reduction in parasitemia were derived by interpolation based on equation 1.

## RESULTS

### *In vitro* potency and *in vivo* pharmacokinetics of the spiroindolone analogs.

A series of chiral spiroindolone analogs were synthesized as part of a lead optimization campaign against P. falciparum (33) (see Fig. S1 in the supplemental material). These compounds were profiled *in vitro* against the human parasite P. falciparum and the rodent parasite P. berghei (Table 2). *In vitro* biological activity was mainly associated with the (+)-enantiomer across the class, suggesting the inhibition of or interaction with a discrete molecular target. Interestingly, the compounds were consistently more potent against P. falciparum than against P. berghei, with a 13- to 27-fold shift in the IC_50_. This finding may be explained by some species selectivity against the ATP4 target and/or differences in the life cycle stage susceptibilities between the two parasites. It is important to mention that although the inactive enantiomers of our most potent analogs [(−)-6 and (−)-7] displayed submicromolar *in vitro* activities against P. falciparum, both compounds showed no efficacy in the P. berghei mouse model. This apparent discrepancy might be explained by the enantiopurity of (−)-6 and (−)-7, which despite having an enantiomer excess (e.e.) of >98% still contained small amounts of the active (more potent) enantiomer as a contaminant.

**TABLE 2:** Summary of *in vitro* potency and *in vivo* pharmacokinetic parameters of spiroindolone analogs following single oral dosing at 25 mg/kg and i.v. dosing at 5 mg/kg to female CD-1 mice^*a*^

Compound^*b*^	IC_50_ (mean ± SD) (nM) for:		Oral PK parameter^*c*^					i.v. PK parameter^*d*^		
	P. falciparum NF54 (*n* = 3)	P. berghei GFP ANKA PbGFP_CON_ (*n* = 3)	*C*_max_ (μg/ml)	*T*_max_ (h)	AUC_0–24_ (μg · h/ml)	*t*_1/2_ (h)^*c*^	*F* (%)	*V*_ss_ (liters/kg)	CL (ml/min/kg)	*t*_1/2_ (h)
(+)-1	13 ± 2.2	242 ± 124	1.2	0.25	1.3	0.7	13	0.9	49.7	0.4
(+)-2	5.6 ± 0.9	75 ± 32	0.6	1	2.9	4.3	62	9.9	92.6	4.6
(+)-3	5.6 ± 0.7	140 ± 54	0.7	2	5.8	6.2	91	13.7	60.1	4.3
(+)-4	5.9 ± 0.9	104 ± 53	1.0	0.25	3.9	5.6	26	1.9	24.0	4.7
(+)-5	4.3 ± 0.4	88 ± 26	3.1	0.25	17.7	4.0	47	1.6	11.8	3.8
(+)-6	0.33 ± 0.06	7.5 ± 2.5	3.1	2	26.8	3.2	53	1.6	8.5	2.9
(+)-7 (KAE609)	1.2 ± 0.2	26 ± 14	0.1^*e*^	1	1.0	8.7	44	2.1	9.7	3.4
			0.25^*e*^	2	2.6	7.5	32			
			3.6	1	43.3	10.0	100			
			10.8^*e*^	24	186.9	9.4	100			
(−)-6	105 ± 20	2,851 ± 897	5	2	68.2	7.9	78	2.8	4.2	13.2
(−)-7	210 ± 19	4,908 ± 1,445	8.3	1	107.3	9.1	92	1.3	2.6	5.6

a IC_50_s were determined at least three times in independent assays (16 h + 8 h of incubation periods, as indicated in Materials and Methods). i.v., intravenous.

b The data in parentheses indicate whether the compound is the (+) or (−) optical isomer.

c *C*_max_, maximum concentration of drug in plasma; *T*_max_, time to *C*_max_; AUC_0–24_, area under the concentration-time curve from 0 to 24 h; *t*_1/2_, elimination half-life; *F*, oral bioavailability.

d *V*_ss_, volume of distribution at steady state; CL, total systemic clearance.

e Dose proportionality PK for KAE609 at 1.5 mg/kg, 5.6, and 105 mg/kg for *C*_max_ values of 0.1, 0.25, and 10.8, respectively, using the formulation 1.1 equimolar 1 N HCl, 5 % Solutol HS15 in 50 mM (pH 3) citrate acid buffer.

A complete set of the *in vitro* PK parameters of all spiroindolone analogs can be found in Table S1 in the supplemental material. The *in vivo* PK parameters obtained following intravenous and oral administration of each compound in CD-1 mice are presented in Table 2. The results reflected a wide range of exposure, with *C*_max_ values from 0.7 μg/ml to 8.3 μg/ml and AUC_0–24_ (area under the concentration-time curve from 0 to 24 h) from 1.3 μg · h/ml to 107.3 μg · h/ml. The volume of distribution at steady state (*V*_ss_) was moderate to high (0.9 to 13.7 liters/kg). The elimination half-life and systemic clearance varied widely and were consistent with the intrinsic clearance measured *in vitro* in liver microsomes. Most analogs showed moderate to high oral bioavailability (26 to 100%), in agreement with the good solubility and permeability observed *in vitro* (Table 2; see also Table S1).

### Dose-response relationship of the spiroindolone analogs in the murine malaria model.

**FIG 1:**
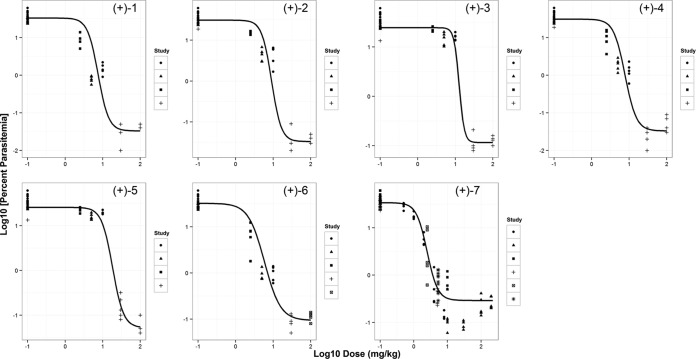
Relationship between dose and parasitemia for spiroindolone analogs. The plot shows the log_10_ parasitemia at day 4 versus log_10_ dose administered in mg/kg for a set of spiroindolone analogs in a P. berghei murine malaria model. A minimum of five dose levels for each compound were used for the dose-response relationship (total doses of 2.5, 5, 10, 30, and 100 mg/kg were administered as a single oral dose at 24 h postinfection). For (+)-7 (KAE609), additional doses ranging between 0.5 and 200 mg/kg were studied. Each data point is from an individual animal, and various symbols correspond to different studies. The solid line represents the predicted profile from the model.

Dose-response experiments were conducted with spiroindolone analogs in the P. berghei mouse model at single oral doses of 2.5, 5, 10, 30, and 100 mg/kg, monitoring parasitemia as primary efficacy readout. Consistent with their poor *ex vivo* potencies against P. berghei, (−)-6 and (−)-7 showed poor efficacy regardless of dose; hence, these two compounds were not included in the current evaluation. Additionally, for several compounds, parasitemia seemed not to change between 30 mg/kg and 100 mg/kg, approaching the detection limit and indicating that the maximum effective concentration had been reached. The relationship between parasitemia and doses was described by a sigmoid dose-response model (Fig. 1). The model parameters, such as *P_max_*, *P_min_*, ED_90_, and Hill slope, were derived and are reported in Table 3. A goodness-of-fit profile for a representative compound [(+)-6] is shown in Fig. S2 in the supplemental material. All the compounds studied displayed a maximum reduction in parasitemia, with ED_90_ ranging from 5.6 (for KAE609) to 38.1 [for (+)-5] mg/kg. KAE609 was identified as the most potent spiroindolone in the series. Interestingly, treatments with none of the spiroindolones except KAE609 resulted in a complete cure (>30 days survival; data not shown).

**TABLE 3:** Dose-response relationship of spiroindolone analogs in the P. berghei malaria mouse model

Compound	*P_max_*^*a*^	*P_min_*^*b*^	ED_90_ (mg/kg)^*c*^	Hill slope
(+)-1	33.11	0.03	15.3	−3.1
(+)-2	30.90	0.03	17.4	−3.36
(+)-3	24.55	0.12	17.9	−6.4
(+)-4	30.90	0.03	16.7	−2.87
(+)-5	25.70	0.05	38.1	−2.99
(+)-6	32.36	0.09	17.3	−2.03
(+)-7 (KAE609)	33.88	0.29	5.6	−2.73

a *P_max_*, maximum parasitemia.

b *P_min_*, minimum parasitemia.

c ED_90_, effective dose causing a 90% reduction in parasitemia.

### KAE609 displays higher exposure in NMRI mice.

The oral PK properties of KAE609 were determined in infected and uninfected NMRI mice at 5.3 mg/kg (Table 4). No significant difference was observed in the PK profiles between the two groups, indicating that the disease status does not affect the PK properties of KAE609. However, at a comparable dose (5 mg/kg), the *C*_max_ and exposure were significantly higher (4-fold) in the NMRI mice than those in the CD-1 mice (Tables 2 and 4). Based on this finding, the concentration-time data in NMRI mice were used for modeling purposes and further PK-PD analysis.

**TABLE 4:** Pharmacokinetic parameters following oral administration of KAE609 to NMRI (uninfected and infected) mice

Mouse infection status	Pharmacokinetic parameter^*a*^							
	*C*_max_ (μg/ml)	AUC_0–∞_ (μg · h/ml)	*V*_max_ (μg/h/kg)	*K_m_* (μg/liter)	*V*_c_ (liters/kg)	*V*_p_ (liters/kg)	*Q* (liters/h/kg)	*K*_a_ (1/h)
Uninfected	1.01	13.21	143	34.1	4.4	5.1	0.61	1.78
Infected	0.96	12.64						

a *C*_max_, maximum concentration of drug reached in plasma; AUC_0–∞_, area under the concentration-time curve from 0 to infinity; CL = *V*_max_/(*K_m_* + *C*_p_), where *C*_p_ is compound plasma concentration, *K_m_* is the compound concentration resulting in 50% clearance saturation, and *V*_max_/*K_m_* is the maximal clearance that can be approximated when *C*_p_ is ≪*K_m_*. The primary model parameters were, CL, plasma clearance; *V*_c,_ central volume of distribution; *V*_p_, peripheral volume of distribution; *Q*, intercompartmental clearance; *K*_a_, absorption rate constant.

### Pharmacokinetic modeling.

**FIG 2:**
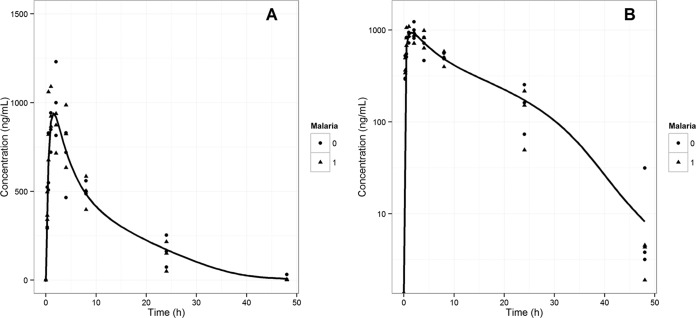
Pharmacokinetics of KAE609 in NMRI mice. Plots represent linear (A) and semilog (B) concentration-time data for KAE609 in infected (0) and uninfected (1) NMRI mice following a single oral dose of 5.3 mg/kg. The line represents the predicted profile from the model.

Nonlinear mixed-effect modeling of the oral PK data led to the estimation of model parameters that might describe the PK behavior of KAE609 in NMRI mice. For the purpose of modeling, a two-compartment PK model with first-order absorption and nonlinear elimination was built in NONMEM. The estimated PK parameters, such as *V*_max_, *K_m_*, *V*_c_, *V*_p_, Q, and *K*_a_, are summarized in Table 4. The linear and semilogarithmic plots of the observed and modeled KAE609 plasma concentrations versus time are shown in Fig. 2A and B, respectively. The goodness of fit was assessed and is depicted in Fig. S3 in the supplemental material.

### KAE609 exhibits time-dependent killing in the P. berghei malaria mouse model.

In the P. berghei malaria mouse model, KAE609 showed 50%, 90%, and 99% reductions in parasitemia after single oral doses of 1.2, 2.7, and 5.3 mg/kg, respectively (32). In order to expand the dynamic range of the pharmacological response, doses ranging from 0.5 to 8 mg/kg were selected for a dose fractionation study. The PK model parameters derived as described above were used to simulate oral plasma concentration-time profiles at different doses and dosing regimens, and the corresponding PK parameters (*C*_max_ and AUC) were calculated using the Berkley Madonna software. The IC_99_ obtained in the *ex vivo* P. berghei maturation assay was estimated (see Materials and Methods) to be 61.6 nM. The 2× the IC_99_ values (123 nM or 48 ng/ml) were used as the threshold (TRE). The PK-PD indices (*C*_max_/TRE, AUC_0–48_/TRE, and %*T*_>TRE_) at different doses and dosing regimens were calculated (see Materials and Methods) and are summarized in Table 1. The results showed that *C*_max_/TRE ranged from 0.4 to 30, AUC_0–48_/TRE ranged from 3 to 514, and finally, the %*T*_>TRE_ was approximately 0 to 100% (Table 1).

**FIG 3:**
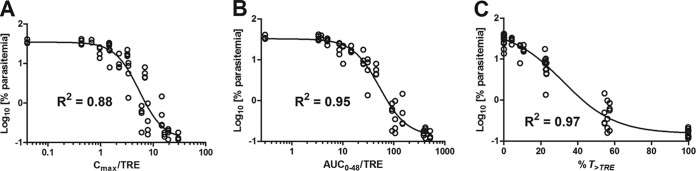
PK-PD relationship for KAE609. The relationships between *C*_max_/TRE (A), AUC_0–48_/TRE (B), and %*T*_>TRE_ (C) of KAE609 and parasitemia, when the total daily dose was administered as a single dose or fractionated in two or three equally divided doses over 24 h, are shown. The line represents the predicted profile from the model.

A sigmoid dose-response model was chosen to describe the data. The relationship between parasitemia and various PK-PD indices is described by equation 1. The derived model parameters, such as *P_max_*, *P_min_*, EC_50_, and Hill slope, for various PK-PD indices (*C*_max_/TRE, AUC_0–48_/TRE, and %*T*_>TRE_) are summarized in Table 5. These results suggest a strong correlation of the efficacy of KAE609 with all three PK-PD indices over a wide range of exposures as a consequence of parameter interdependency. Among the three indices, the percentage of the time that the KAE609 plasma concentration remained at >2× the IC_99_ (%*T*_>TRE_) and the AUC_0–48_/TRE correlated best (*R*^2^ = 0.97 and 0.95, respectively) with a reduction in parasitemia, followed by the *C*_max_/TRE (*R*^2^ = 0.88) (Fig. 3). Further, %*T*_>TRE_ displayed the lowest magnitude for the standard error of the estimate (standard deviation of the regression [Sy.x], 0.17), followed by AUC_0–48_/TRE (0.22) and *C*_max_/TRE (0.34). Collectively, our data indicate a trend favoring time over threshold and exposure over threshold as most important determinants of efficacy for KAE609 rather than the concentration over threshold.

**TABLE 5:** PK-PD model parameters for KAE609

Parameter^*a*^	Value (95% CI) for^*b*^:		
	*C*_max_/TRE	AUC_0–48_/TRE	%*T*_>TRE_
*P_min_*	0.12 (0.05 to 0.29)	0.13 (0.09 to 0.20)	0.15 (0.12 to 0.20)
*P_max_*	34.51 (21.78 to 54.58)	32.66 (25.06 to 42.56)	53.09 (32.28 to 87.50)
EC_50_	5.43 (4.2 to 7.0)	52.72 (44.16 to 62.95)	32.06 (28.04 to 36.07)
Hill slope	−1.87 (−2.69 to −1.05)	−1.63 (−2.04 to −1.22)	−0.031 (−0.037 to −0.024)
*R*^2^	0.88	0.95	0.97

a *P_max_*, maximum parasitemia; *P_min_*, minimum parasitemia, EC_50_, value required to produce 50% of the maximal effect.

b CI, confidence interval; *C*_max_/TRE, ratio of peak plasma concentration (*C*_max_) to the threshold (TRE = 2× the IC_99_); AUC_0–48_/TRE, ratio of the area under the concentration-time curve from 0 to 48 h (AUC_0–48_) to the threshold; %*T*_>TRE_, percentage of the 48-h period during which the total compound concentration exceeded the threshold.

## DISCUSSION

In the field of anti-infectives, the doses and regimens selected in clinical trials are increasingly informed by dose fractionation studies in animals (9, 10). This approach was not traditionally adopted for antimalarial drugs, as most agents were developed when therapeutic strategies were based largely on empirical clinical evidence. Recently, PK-PD relationships have been established for chloroquine (time-dependent killing) and artesunate (concentration-dependent killing) (27). This study is an attempt to understand the dose-response relationships for a class of spiroindolone analogs and to identify the PK-PD driver of the reduction in parasitemia resulting from treatment with KAE609. The results presented in this study must be interpreted with a few limitations in mind. First, the single-dose PK parameters determined in the NMRI mice were assumed to be similar to the multiple-dose PK parameters and were correlated with the efficacy data for KAE609. Second, the PK data used for PK-PD analysis at different doses and regimens were simulated based on the model parameters from single-dose PK under the assumption of nonlinearity observed in both the NMRI and CD-1 mice. The third limitation of this study is that in the absence of supporting intravenous PK data in NMRI mice, oral bioavailability was assumed to be 100%.

A class of spiroindolones was identified in a whole-cell screening against the human malaria parasite P. falciparum. In order to support the selection of compounds to progress in the murine malaria model, the *in vitro* potency was also assessed against P. berghei (rodent parasite). A systematic 13- to 27-fold shift in IC_50_ was observed between P. falciparum and P. berghei for all study compounds (Table 2). The likely explanations for the differential sensitivities to the spiroindolones might be either species susceptibility differences (P. falciparum versus P. berghei) or stage selectivity. It should indeed be noted that the P. falciparum assay covers intracellular parasite growth and reinfection of new blood cells, with growth inhibition measured after 24 h. In contrast, the P. berghei *in vitro* (*ex vivo*) assay is a schizont maturation assay over a single life cycle without reinvasion of new red blood cells over a 24-h period.

The dose-response experiments were performed in mice with a series of potent spiroindolones. Parasitemia was used as the primary pharmacodynamic (PD) readout for multiple reasons. It provides a wide dynamic range and is a fast, easy, and reproducible measurement. Unfortunately, in this P. berghei model, a real-time measurement of parasitemia over time that could be used to determine the more clinically relevant parasitemia reduction ratio (PRR) was not feasible. For each compound, the data derived from all experiments (i.e., doses) were fitted simultaneously using nonlinear mixed-effect modeling (using NONMEM VI). A good correlation was observed between the doses and a reduction in parasitemia across the spiroindolone class (Fig. 1 and Table 3), with ED_90_s ranging from 6 to 38 mg/kg. These results are in line with those available for the currently used antimalarials in the P. berghei ED_90_-normalized assay (31). Generally, the investigated compounds displayed a rather steep Hill slope (≥2). Specifically, for compounds (+)-3 and (+)-5, due the limited data points covering the middle portion of the dose-response curves, our confidence in the derived Hill slopes is moderate. Interestingly, all the spiroindolone analogs tested achieved a complete reduction in parasitemia (below the detection limit, <0.1%), but only KAE609 showed a complete cure (i.e., survival > 30 days). The reason underlying this observation is yet to be elucidated.

The most promising spiroindolone, KAE609, which is currently in clinical development, was selected for more extensive dose fractionation studies in the P. berghei murine model. The objective of such an investigation was to identify the PK-PD driver of efficacy and determine whether this compound exhibits time- or concentration-dependent killing. The results suggested that the time during which plasma concentrations remained at >2× the IC_99_ (%*T*_>TRE_) and the AUC_0–48_/TRE correlated slightly better (although not statistically significantly) with a reduction in parasitemia than did *C*_max_/TRE. In our study, all three PK-PD indices (*C*_max_/TRE, AUC_0–48_/TRE, and %*T*_>TRE_) correlated well with a reduction in parasitemia as a consequence of their significant interdependency (Spearman correlation coefficient, >0.97; *P* value < 0.0001). The PK properties of KAE609 (i.e., long half-life) and the almost complete reduction in parasitemia reached in a narrow dose range do not allow us at this stage to clearly distinguish between concentration- and time-dependent killing for KAE609 in the investigated model. A definitive conclusion might be achieved by a more extensive dose fractionation study up to 48 h with several different dosing regimens to break the colinearity (44–46). Based on the correlation analysis and the standard error of the estimate, collectively, our data indicate a trend favoring time over threshold and exposure over threshold as most important determinants of efficacy. This would suggest time-dependent rather than concentration-dependent killing for KAE609. In addition, a conservative interpretation of the results is the prediction that a dosing regimen covering a *C*_max_/TRE of 30, AUC_0–48_/TRE of 587, and *T*_>TRE_ of 100% for an observation period of 48 h is likely to yield a maximum reduction in parasitemia (parasitemia, <0.1%) in the malaria mouse model. Interestingly, the concentration of KAE609 needed to inhibit 50% of the parasites (P. berghei) *in vitro* (IC_50_, 26 nM or 10 ng/ml; Table 2) is significantly lower than the one needed to reduce parasitemia by 50% *in vivo* (*C*_max_, 260 ng/ml), as derived from *C*_max_/TRE (Table 5). The difference might be due to the limitations of the *in vitro* experimental setting, which does not capture reinvasion. Also, parasites are exposed to a constant concentration of the compound over time *in vitro* (static system), whereas *in vivo* (dynamic system), the exposure varies with time, according to the pharmacokinetics of the compound. In addition, the protein binding might differ in the two systems (47). KAE609 and other spiroindolone analogs showed very high plasma protein binding in mice (≥99%) (see Table S1 in the supplemental material). It is challenging to accurately measure the free fraction for highly protein-bound compounds (48). For our analysis, we used total plasma concentrations when analyzing the PK-PD relationship, similarly to what was reported for other drug candidates, such as bedaquiline (49). Considering the relationship between *in vitro* potency and concentration achieved in mice, compounds with a *C*_max_/TRE of ≥30, AUC_0–48_/TRE of ≥587, and *T*_>TRE_ of 100% would likely be efficacious and would be prioritized for further studies.

The use of murine models in drug discovery for both pharmacokinetic and pharmacodynamic assessment of antimalarial test compounds is widespread (31, 32, 50, 51). These models can generate robust PK-PD data that can be used for dose optimization. This has been reported for dihydroartemisinin (DHA) (52), piperaquine (53), and chloroquine (54). The data reported in this publication were generated in either uninfected CD-1 mice (PK) or NMRI mice (PD). Surprisingly, for KAE609, significant strain differences were observed, yielding a 4-fold higher exposure in NMRI mice compared to that in CD-1 mice. No significant difference between the infected and uninfected NMRI mice was observed; these results are consistent with published reports showing no impact of malaria infection on the PK of antimalarial agents (53–55). The reasons for the observed strain difference are yet to be understood. A two-compartment pharmacokinetic model with nonlinear elimination was chosen to fit the plasma concentration versus time data of KAE609 generated in NMRI mice (infected and uninfected) at 5.3 mg/kg. The nonlinear behavior for KAE609 was previously observed in the dose proportionality studies in CD-1 mice (Table 2; see Fig. S4 in the supplemental material). The nonlinear mixed-effects modeling provides a good solution for modeling sparse data sets and has been well established in preclinical and clinical situations (56). The concentration-time data obtained from different populations (infected and uninfected NMRI mice) were fitted simultaneously using this method. The estimated model parameters were used to simulate the PK profiles of KAE609 at any of the doses and regimens administered in the dose fractionation study.

In conclusion, all spiroindolone analogs studied displayed a good dose-response relationship in the P. berghei murine model. KAE609 exhibits a trend favoring time or exposure over threshold as most important determinants of reduction in parasitemia. Furthermore, our results suggest that for KAE609 and, supposedly, for its analogs, dosing regimens covering a *T*_>TRE_ of 100%, AUC_0–48_/TRE of 587, and *C*_max_/TRE of 30 are likely to result in the maximum reduction in parasitemia (<0.1%) in the P. berghei mouse model of malaria. Based on the present results, the optimization campaign of leads belonging to the same chemical class could be guided by a PK-PD driven strategy. Compounds could be characterized in terms of their potencies and *in vivo* pharmacokinetics. PK-PD indices could be estimated and compared to those derived here for KAE609. Compounds showing promising properties (i.e., matching the indices of KAE609) could be prioritized and efficacy studies designed in an informed manner in order to maximize the reduction in parasitemia. A similar approach could be used as a guide for designing clinical studies. Assuming similar PK-PD relationships, regardless of the parasite species, the PK-PD indices (as a TRE, the human-relevant 2 × P. falciparum IC_99_ is to be considered) derived in the present study could be applied to the clinical situation. Our results support a recent clinical phase II study with KAE609 demonstrating positive results with a 3-day dosing regimen of 30 mg daily clearing parasitemia in P. vivax and P. falciparum malaria patients (57). The average PK parameters from the two cohorts were a *C*_max_ of 855 ng/ml, AUC of 14,300 ng · h/ml, and % *T*_>TRE_ of 100% (2 × P. falciparum IC_99_ = 1 ng/ml = TRE). Such parameters are comparable to the ones derived for KAE609 in the P. berghei murine malaria model (*C*_max_, 1,440 ng/ml; AUC, 28,176 ng · h/ml; %*T*_>TRE_, 100% [2× the P. berghei IC_99_ = 48 ng/ml = TRE]). This demonstrates that the PK-PD indices required for a reduction in parasitemia were similar between the mouse and human models. Overall, our results could be used to prioritize analogs within the same class of compounds and contribute to the design of efficacy studies, thereby facilitating early drug discovery and development programs.

## Supplementary Material

Supplemental material
