# Supplementary material for: Pharmacokinetic-Pharmacodynamic Analysis of Spiroindolone Analogs and KAE609 in a Murine Malaria Model
Source: Antimicrob Agents Chemother. 2015 Jan 27;59(2):1200–10. doi: 10.1128/AAC.03274-14 (PMC4335872; doi:10.1128/AAC.03274-14)
Supplement: Supplemental material [file supp_59_2_1200__index.html]

Pharmacokinetic-Pharmacodynamic Analysis of Spiroindolone Analogs and KAE609 in a Murine Malaria Model — Supplemental material 

# Pharmacokinetic-Pharmacodynamic Analysis of Spiroindolone Analogs and KAE609 in a Murine Malaria Model

## Supplemental material

**Files in this Data Supplement:**

- Supplemental file 1 -

  Table S1 and Fig. S1 to S4.

  PDF, 350K
