# Supplementary material for: Pharmacokinetic-Pharmacodynamic Analysis of Spiroindolone Analogs and KAE609 in a Murine Malaria Model
Source: Antimicrob Agents Chemother. 2015 Jan 27;59(2):1200–10. doi: 10.1128/AAC.03274-14 (PMC4335872; doi:10.1128/AAC.03274-14)
Supplement: Supplemental material [file AAC.03274-14_zac002153716so1.pdf]

# Pharmacokinetics-pharmacodynamics analysis of spiroindolone analogs and KAE609 in a murine malaria model

Suresh B. Lakshminarayana[1#], Céline Freymond[2,3], Christoph Fischli [2,3], Jing Yu[4], Sebastian Weber[5], Anne Goh[1], Bryan K. S. Yeung[1], Paul C. Ho[6], Véronique Dartois[1,a], Thierry T. Diagana[1], Matthias Rottmann[2,3] and Francesca Blasco[1#]

[(1)]Novartis Institute for Tropical Diseases, Singapore

[(2)]Swiss Tropical and Public Health Institute, Basel, Switzerland

[(3)]University of Basel, Switzerland

[(4)]Novartis Institutes for Biomedical Research, Cambridge, MA, USA

[(5)]Novartis Pharma, Basel, Switzerland

[(6)]Department of Pharmacy, National University of Singapore, Singapore

[#]Corresponding Authors SBL & FB

Suresh B. Lakshminarayana

Novartis Institute for Tropical Diseases, 10 Biopolis Road, #05-01 Chromos

Singapore, 138670

Email: suresh.b_lakshminarayana@novartis.com; Phone: +65 67222991

Francesca Blasco

Novartis Institute for Tropical Diseases, 10 Biopolis Road, #05-01 Chromos

Singapore, 138670

Running title: Preclinical PK-PD of antimalarials

Email: francesca.blasco@novartis.com; Phone: +65 67222967

Present address:

[(a)]Public Health Research Institute Center, New Jersey, USA

## Supplementary material

**Table S1.** *In vitro* PK properties of spiroindolone analogs

**Figure S1:** Structure of spiroindolone analogs

**Figure S2.** Goodness-of-fit plots for dose response relationship

**Figure S3.** Goodness-of-fit plots for pharmacokinetic modeling of KAE609

**Figure S4.** Dose proportionality for KAE609 in CD-1 mouse

**Table S1:** *In vitro* PK properties of spiroindolone analogs

| Compound | Solubility (pH 6.8, µM) [a] | PAMPA[b] %FA | Caco-2[c] $P_{app}$(A-B) $10^{-6}$ cm/sec | Hepatic $CL_{int}$ in mouse liver microsomes[d] (µL/min/mg) | Mouse PPB[e] (%) | *RBC to Plasma Ratio*[f] |
|---|---|---|---|---|---|---|
| (+)-**1** | 194 | 97 | 7.19 | 770 | 98.4 | 1.04 |
| (+)-**2** | 22 | 98 | n.t | 92 | 99.8 | 0.77 |
| (+)-**3** | 8 | 98 | 41.7 | 35 | 99.7 | 0.93 |
| (+)-**4** | 137 | 97 | 7.66 | 330 | 98.8 | 0.62 |
| (+)-**5** | 131 | 98 | 4.33 | 26 | 98.5 | 1.18 |
| (+)-**6** | 169 | 99 | 5.55 | 41 | 99.5 | 1.12 |
| (+)-**7** (KAE609) | 39 | 98 | 1.69 | 28 | 99.8 | 0.85 |
| (-)-**6** | 112 | 97 | 4.8 | 36 | 99.6 | 0.81 |
| (-)-**7** | 35 | 98 | 1.73 | 21 | 99.4 | 0.52 |

*In vitro* PK parameters like solubility, PAMPA, Caco-2 permeability, hepatic clearance, mouse plasma protein binding and RBC to plasma partitioning were determined in a medium

to high through put format using in-house standard assay protocols that are described in the literature.

[(a)]HT-Equilibrium solubility at pH 6.8 (1); [(b)]Parallel Artificial Membrane Permeability Assay expressed in % fraction absorbed (FA) (2, 3); [(c)]Apparent permeability from apical to basolateral in Caco-2 cells expressed as $P_{app}$ (A-B) $10^{-6}$ cm/sec (4, 5), n.t, not tested; [(d)]intrinsic clearance in mouse liver microsomes expressed as µL/min/mg protein, in the presence of liver enzymes (6); [(e)]Mouse plasma protein binding using rapid equilibrium dialysis method (7, 8), [(f)]RBC to plasma ratio, mean from 1 µM & 10 µM (9).

**Figure S1**. Structure of spiroindolone analogs.

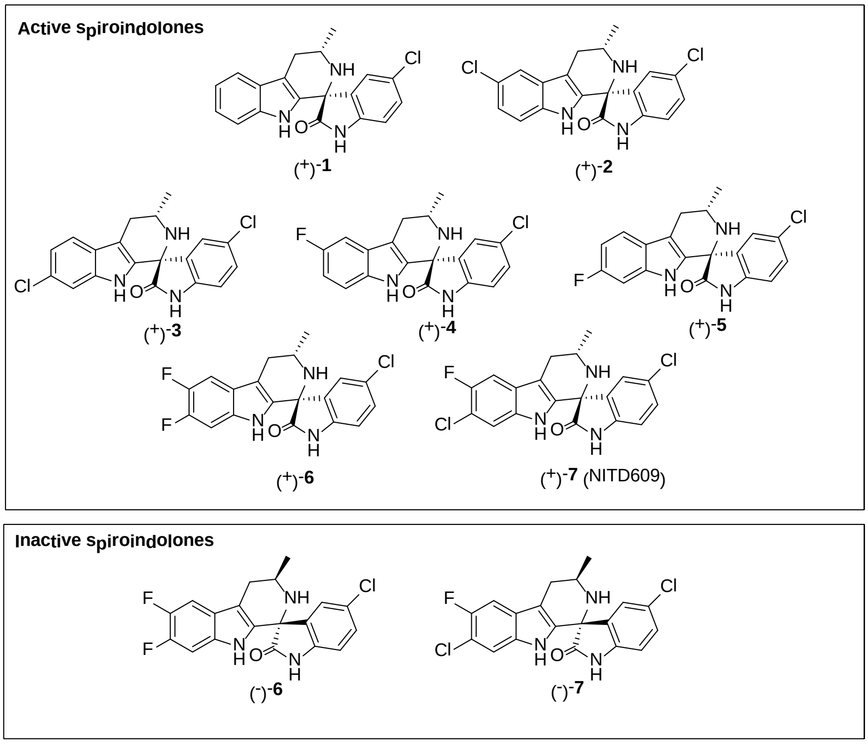


Active spiroindolones have the 1R, 3S configuration while inactive spiroindolones have the 1S, 3R configuration (10).

**Figure S2.** Goodness-of-fit plots for dose response relationship

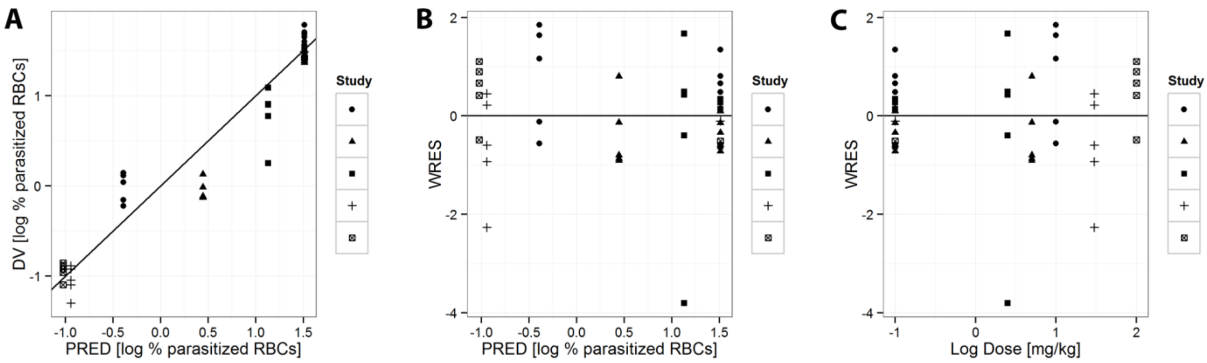


Goodness-of-fit plots for a representative compound [(+)-6] (A) observed data (DV) vs populations predictions (PRED) (B) weighted residuals (WRES) vs PRED (C) WRES vs Dose

**Figure S3.** Goodness-of-fit plots for pharmacokinetic modeling of KAE609

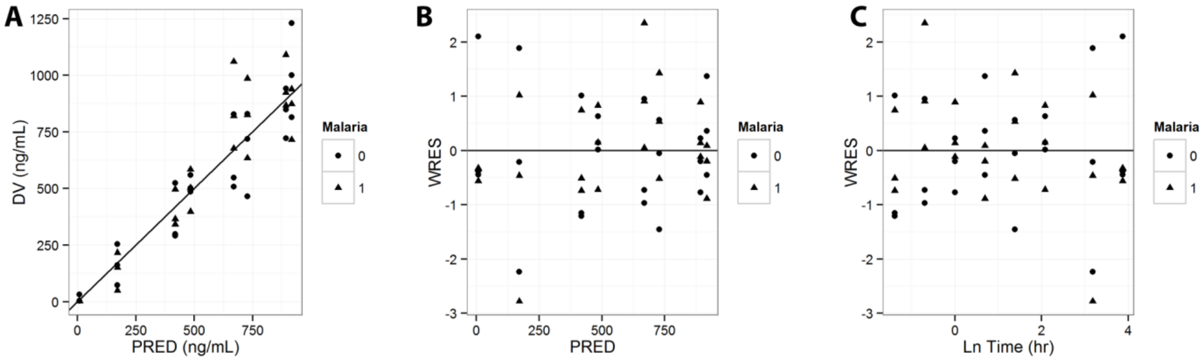


Goodness-of-fit plots for KAE609 pharmacokinetic modeling (A) observed data (DV) vs populations predictions (PRED) (B) weighted residuals (WRES) vs PRED (C) WRES vs Time

**Figure S4.** Dose proportionality for KAE609 in CD-1 mouse

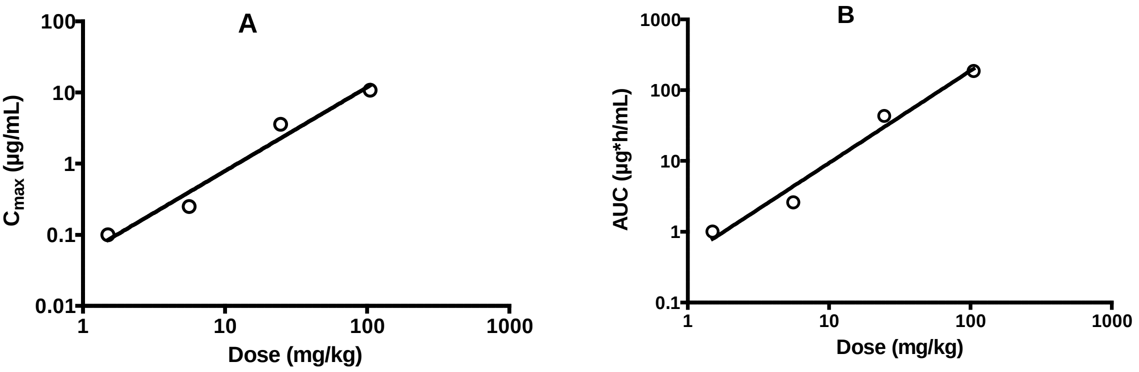


Dose linearity tests on $C_{max}$ and $AUC_{0\text{-}24}$ were carried out by the regression of log-transformed data (power regression model) (11, 12). Doses (1.5 – 105 mg/kg) and PK parameters ($C_{max}$ and $AUC_{0\text{-}24}$) were log-transformed, and correlation coefficient ($R^2$), slope, and 95 % confidence intervals of slope were calculated using the GraphPad Prism version 5.02 for windows (GraphPad software, San Diego, California USA). The system was considered to be linear when $R^2 \sim 1$, Slope 95 % $CI_{lower} \geq 0.8$, and $CI_{upper} \leq 1.25$ (11, 12). (A) Dose vs $C_{max}$ ($R^2 = 0.97$, Slope = 1.18, 95 % $CI_{lower} = 0.52$ and $CI_{upper} = 1.83$); (B) Dose vs $AUC_{0\text{-}24}$ ($R^2 = 0.97$, Slope = 1.30, 95 % $CI_{lower} = 0.65$ and $CI_{upper} = 1.96$). Based on the above observations, KAE609 shows nonlinear behavior in CD-1 mouse between the doses studied.

## Reference List


1. **Zhou, L., L. Yang, S. Tilton, and J. Wang.** 2007. Development of a high throughput equilibrium solubility assay using miniaturized shake-flask method in early drug discovery. J.Pharm.Sci. **96**:3052-3071. doi:10.1002/jps.20913 [doi].

2. **Avdeef, A.** 2005. The rise of PAMPA. Expert.Opin.Drug Metab Toxicol. **1**:325-342. doi:10.1517/17425255.1.2.325 [doi].

3. **Avdeef, A., S. Bendels, L. Di, B. Faller, M. Kansy, K. Sugano, and Y. Yamauchi.** 2007. PAMPA--critical factors for better predictions of absorption. J.Pharm.Sci. **96**:2893-2909. doi:10.1002/jps.21068 [doi].

4. **Balimane, P. V. and S. Chong.** 2005. Cell culture-based models for intestinal permeability: a critique. Drug Discov.Today **10**:335-343. doi:S1359644604033549 [pii];10.1016/S1359-6446(04)03354-9 [doi].

5. **Marino, A. M., M. Yarde, H. Patel, S. Chong, and P. V. Balimane.** 2005. Validation of the 96 well Caco-2 cell culture model for high throughput permeability assessment of discovery compounds. Int.J.Pharm. **297**:235-241. doi:S0378-5173(05)00186-9 [pii];10.1016/j.ijpharm.2005.03.008 [doi].

6. **Obach, R. S.** 1999. Prediction of human clearance of twenty-nine drugs from hepatic microsomal intrinsic clearance data: An examination of in vitro half-life approach and nonspecific binding to microsomes. Drug Metab Dispos. **27**:1350-1359.

7. **Banker, M. J., T. H. Clark, and J. A. Williams.** 2003. Development and validation of a 96-well equilibrium dialysis apparatus for measuring plasma protein binding. J.Pharm.Sci. **92**:967-974. doi:10.1002/jps.10332 [doi].

8. **Kariv, I., H. Cao, and K. R. Oldenburg.** 2001. Development of a high throughput equilibrium dialysis method. J.Pharm.Sci. **90**:580-587. doi:10.1002/1520-6017(200105)90:5<580::AID-JPS1014>3.0.CO;2-4 [pii].

9. **Yu, S., S. Li, H. Yang, F. Lee, J. T. Wu, and M. G. Qian.** 2005. A novel liquid chromatography/tandem mass spectrometry based depletion method for measuring red blood cell partitioning of pharmaceutical compounds in drug discovery. Rapid Commun.Mass Spectrom. **19**:250-254. doi:10.1002/rcm.1777 [doi].

10. **Yeung, B. K., B. Zou, M. Rottmann, S. B. Lakshminarayana, S. H. Ang, S. Y. Leong, J. Tan, J. Wong, S. Keller-Maerki, C. Fischli, A. Goh, E. K. Schmitt, P. Krastel, E. Francotte, K. Kuhen, D. Plouffe, K. Henson, T. Wagner, E. A. Winzeler, F. Petersen, R. Brun, V. Dartois, T. T. Diagana, and T. H. Keller.** 2010. Spirotetrahydro beta-carbolines (spiroindolones): a new class of potent and orally efficacious compounds for the treatment of malaria. J.Med.Chem. **53**:5155-5164. doi:10.1021/jm100410f [doi].

11. **Hummel, J., S. McKendrick, C. Brindley, and R. French.** 2009. Exploratory assessment of dose proportionality: review of current approaches and proposal for a practical criterion. Pharm.Stat. **8**:38-49. doi:10.1002/pst.326 [doi].

12. **Smith, B. P., F. R. Vandenhende, K. A. DeSante, N. A. Farid, P. A. Welch, J. T. Callaghan, and S. T. Forgue.** 2000. Confidence interval criteria for assessment of dose proportionality. Pharm.Res. **17**:1278-1283.
